# The Degradation of Daguerreotypes and the Relationship with Their Multi-Material Structure: A Multimodal Investigation

**DOI:** 10.3390/s23094341

**Published:** 2023-04-27

**Authors:** Diego Quintero Balbas, Barbara Cattaneo, Andrea Cagnini, Paolo Belluzzo, Sandra Rossi, Raffaella Fontana, Jana Striova

**Affiliations:** 1 National Research Council-National Institute of Optics (CNR-INO), Largo E. Fermi 6, 50125 Florence, Italy; diegoivan.quinterobalbas@ino.cnr.it (D.Q.B.); raffaella.fontana@ino.cnr.it (R.F.); 2 Laboratorio di Restauro Cartacei e Membranacei, Opificio delle Pietre Dure–MiC, Viale F. Strozzi, 1, 50129 Firenze, Italy; barbara.cattaneo@cultura.gov.it (B.C.); sandra.rossi@cultura.gov.it (S.R.); 3 Laboratorio Scientifico, Opificio delle Pietre Dure–MiC, Viale F. Strozzi, 1, 50129 Firenze, Italy; andrea.cagnini@cultura.gov.it; 4 Laboratorio di Restauro Oreficerie, Opificio delle Pietre Dure–MiC, Via degli Alfani 78, 50121 Firenze, Italy; paolo.belluzzo@cultura.gov.it

**Keywords:** daguerreotypes, non-invasive sensing, vibrational spectroscopy, corrosion, optical instrumentation, Cu formates, metal carboxylates, OCT, XRF

## Abstract

Preserving and analytically examining daguerreotypes is particularly challenging because of their multi-material and multi-component structure. Various sensors have been exploited to examine mainly the image plates of the daguerreotypes even though the degradation goes beyond this component. Micro-analyses have been the preferred method due to the nanoscale structure of the image particles. In this work, we propose comprehensive multi-modal non-invasive sensing to investigate the corrosion products present in nine daguerreotypes from the Fondazione Alinari per la Fotografia (FAF, Florence, Italy). The methodology proposed includes chemical and morphological analyses: portable X-ray fluorescence spectrometry (pXRF), Raman microspectroscopy (μ-Raman), and micro-Fourier transform infrared spectroscopy in reflection mode (μ-rFTIR) for the chemical identification. For the first time, optical coherence tomography (OCT) was deployed to record the cross-sectional and morphological data of the relevant corrosion formations on daguerreotypes in a contactless way. The results allowed the characterization, in a non-invasive mode at a microscopic level, of a wide range of degradation products produced by the interaction of the different elements present in the structure of the daguerreotypes. The aim was to verify the performance of the proposed methodology and to link the chemical and physical complexity of the entire structure, disclosed by the state-of-art sensors, to the daguerreotype degradation. The results draw attention to the need to monitor not only the image condition but the whole object as a partially closed system in constant interaction internally and with the environment.

## 1. Introduction

Daguerreotypes—obtained with the first commercially available photographic process—are complex unique objects that are fragile and prone to physical and chemical degradation. In particular, corrosion may be induced by the chemical interaction with the environment, with degradation by-products from the various materials present in their structure, or previous attempts of conservation [1].

**Figure 1:**
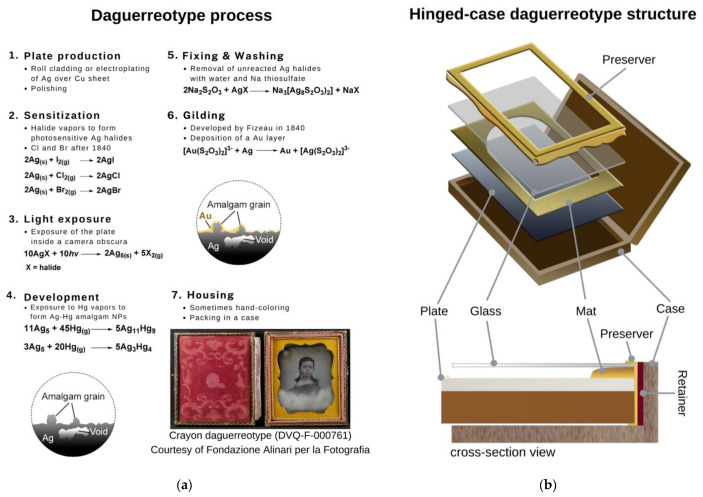
(**a**) The steps of the process with the listed chemical reactions. The image of DVQ-F-000761, courtesy of Fondazione Alinari per la Fotografia, is shown in step 7. (**b**) Scheme of the structure of a hinged-case daguerreotype with the different elements indicated to show its multi-material complexity.

The process to realize a daguerreotype—contrived by Niépce and Daguerre and later modified to improve its effectiveness—consists of several steps summarized in Figure 1a [2,3,4,5]. The image particles, with a nanoscale dimension, are formed over the copper-silver plate. The nanoparticles (NPs) are constituted by Ag-Hg amalgams (e.g., the schachnerite ζ phase (hexagonal Ag_11_Hg_9_) and γ phase (body-centered cubic Ag_3_Hg_4_)) [1,3,4,6] produced after a series of chemical reactions [1,3]. Because of their intrinsic fragility, the daguerreotypes were enclosed in a package (step 7), made of various materials (e.g., metals, textiles, glass, and wood) [7], as schematized in Figure 1b. Sometimes, before its housing, daguerreotypes were colored manually with colorants and binders applied over the plate surface ([8] and references herein).

The analytical examination of the daguerreotypes is particularly challenging due to their multi-material and multi-component structure. In particular, Table 1 shows evidence of the most commonly applied sensing technology to reveal their degradation. The degradation of the image plate can be fast and irreversible [9]. Its main tarnishing product is silver sulfide—acanthite (Ag_2_S)—and to a lower extent other Ag compounds, such as Ag oxides or Au-Ag sulfides (e.g., AuAgS or AuAg_3_S_2_), the latter in gilded daguerreotypes [10,11]. Some daguerreotypes are particularly photosensitive as a consequence of the formation of AgCl in the image particles. Chlorine, which can also damage the Ag-Cu plate, can be a residue of the manufacturing process, the application of historical cleaning treatments (e.g., thiourea solutions acidified with HCl), or the interaction with atmospheric pollutants [2]. In addition, some early cleaning methodologies may induce modifications to the image NPs morphology and activate corrosion mechanisms, for example, KCN cleaners, which could produce silver cyanide (AgCN), while thiourea solutions could cause etching, retarnishing, and Ag phosphate (Ag_3_PO_4_) deposition (as a result of the addition of phosphoric acid for pH adjustment) [1,12,13]. The extreme fragility of the objects (physically and photochemically) and the ultrafine structure of the image calls for the techniques with high spatial/spectral resolution sensors that enable obtaining information in a non-invasive way.

As summarized in Table 1, up to now, microscopic sensing (e.g., confocal and different modalities of electron microscopy [1,3,14,15], micro-Raman [7,16], and occasionally Synchrotron-based analysis [10]) was exploited for studying daguerreotypes, obtaining both morphological and chemical information from the image surface. However, the size of the plate or the invasiveness (required physical sampling) might limit their application. The microscopic investigations shed light on the image particle formation, on their shape, and how their modifications impact the optical properties of the daguerreotype [3,5,6]. Additionally, elemental spot sensing with X-ray fluorescence (XRF) [10] and Laser-Induced Breakdown Spectroscopy (LIBS) [17] proved particularly useful due to the prevalence of the metallic and glass elements inside the daguerreotypes structures; however, the latter is micro-invasive. Molecular sensing, namely Raman spectroscopy, can exploit the nanostructure of the daguerreotype plate to produce a surface-enhanced Raman spectroscopy (SERS) effect [18], yet the laser power must be kept low to avoid the photoreduction of Ag. Other vibrational sensing, through attenuated total reflectance Fourier transform infrared spectroscopy (ATR-FTIR), has limited applicability due to the required sampling or direct contact with the material [19]. Imaging methods, such as technical photography (ultraviolet (UV) and visible) hyperspectral reflectance and XRF mapping, focus mainly on the daguerreotype plate [10,17,18,20] to register its characteristics [11,21,22,23]. 

**Table 1:** Degradation products disclosed by different sensing technology in daguerreotypes as reported in the literature.

Section	Degradation Product	Analytical Technique	Lit.
Glass	“Weeping” (sodium silicate gels)	SEM-EDS, X-ray Diffraction (XRD), FTIR-ATR (after sampling)	[19,24]
	(Na, K) Sulfates		
	Formates		
Mat and Preserver	Na/Cu formates and carbonates	Raman	[25,26]
Image Plate	Acanthite (Ag2S), silver sulfate, silver chloride, silver oxide	SEM-EDS, XRD, Raman, Synchrotron-based techniques, LIBS	[1,2]
	Cu oxide, Cu sulfide		

In this respect, we propose a comprehensive non-invasive, multi-sensorial protocol to examine the complete multi-material structure of daguerreotypes at a microscale, with special regard to the corrosion products. The methodology consists of elemental and molecular sensing through portable X-ray fluorescence spectrometry (pXRF), Raman microspectroscopy (μ-Raman), and micro-Fourier transform infrared spectroscopy in reflection mode (μ-rFTIR) for the chemical identification and optical coherence tomography (OCT) for the contactless, cross-sectional, and morphological characterization. The results obtained on nine daguerreotypes from the Fondazione Alinari per la Fotografia (FAF, Florence, Italy) demonstrate the versatility of such a combination of the sensors to disclose the corrosion products on a microscale.

## 2. Materials and Methods

### 2.1. Daguerreotypes Studied

Nine daguerreotypes from the Fondazione Alinari per la Fotografia (FAF) from the Tuscany Region (Florence, Italy) [27] were examined. Table 2 summarizes the main information about each object.

### 2.2. Portable X-ray Fluorescence Spectrometry

X-ray fluorescence analyses were performed with a portable XGLab ELIO spectrometer with an X-ray tube with a Rh anode coupled to a 1 mm collimator. The measurements were performed using 50 kV, 40 mA, and 120 s. The instrument was equipped with a 25 mm^2^, Peltier-cooled, Si Drift Detector (energy resolution < 135 eV on the Mn-kα line at 0.5 ms shaping time, a peak-to-background ratio of the order of 15,000, silicon thickness = 500 mm, Be window thickness = 12 μm). The focal distance from the detection head (90°/63.5° measurement/detection geometry) was 1.4 cm, and the spatial resolution was ~1.2 mm. The intensity, expressed in counts per second (cps), was calculated by dividing the area under the correspondent characteristic analytical line in the XRF spectra by the measuring time in seconds. The data were processed with Elio SW and PyMCA software.

### 2.3. Raman Microspectroscopy

Raman microspectroscopy (μ-Raman) spectra were measured with a Renishaw inVia Raman confocal microscope equipped with a Leica DM2700 optical microscope and two excitation sources at 532 nm and 785 nm. The measurements were performed in an extended spectral range of 100–3200 cm^−1^ with a grating of 1800 L/mm for 532 nm and 1200 L/mm for 785 nm and a thermoelectrically cooled CCD detector (spectral range 400–1060 nm) with a spectral resolution of 1 cm^−1^ per CCD pixel (functional resolution of 3 cm^−1^). The laser power on the daguerreotype surface was <1 mW, with typical 10–20 s integration times and 1–5 accumulations. The data spectra were collected with 50× (N PLAN NA = 0.5; theoretical spot size_532_ = 0.65 μm and spotsize_785_ = 0.95 μm) long-distance objective and processed with WiRE™ 5.5 (Renishaw plc.) and Origin® Pro 8.5 (OriginLab Corporation) software.

The Raman bands of Ag sulfide compounds were fitted by the proper number of Gaussian curves to better determine the band positions with Origin® Pro 8.5 (OriginLab Corporation).

### 2.4. Micro Reflection Fourier Transform Infrared Spectroscopy

Micro reflection Fourier transform infrared (μ-rFTIR) spectra were acquired in a contactless reflection mode with a Thermo Nicolet Continuum microscope equipped with a 15× objective (NA 0.58). The spectra were recorded in the 4000–400 cm^−1^ spectral range, with a spectral resolution of 4 cm^−1^ and the autogain mode activated. To maximize the signal-to-noise ratio, each measurement was performed with an aperture of 175 × 175 μm^2^ and 256 scans. The background acquisition was performed with the same conditions over a Au-coated glass slide. The data were processed with Omnic 9.0 (Thermo Fisher Scientific Inc.) and Origin Pro 8.5 software.

### 2.5. Spectral-Domain Optical Coherence Tomography

We performed morphological and cross-sectional analyses of resin layers and corrosion deposits with a commercial optical coherence tomography (OCT) device (Thorlabs Ganymede). We used a superluminescent diode with a central wavelength of 900 nm and a bandwidth of about 150 nm. The axial resolution in the air was 3 μm, while the lateral resolution was 4 μm. The detector consisted of a spectrograph made of a diffraction grating and a fast camera, all controlled via 64-bit ThorImage® 5.0 software (Thorlabs Inc.) preinstalled on a high-performance computer.

The 3D scanning path probe with an integrated video camera performs high-speed imaging (76 kHz) for rapid volume acquisition and live display. For the OCT cross-section, the acquisition field of view (FOV) was 2 × 1 mm^2^, with a XZ pixel size of 2 × 1.96 μm^2^. For the 3D image cube reported here, the FOV was XYZ 2 × 2 × 1 mm^3^, with pixel size of 2 × 2 × 1.96 μm^3^.

## 3. Results

### 3.1. Characterization of the Multi-Material Structure: Plate, Mat, Preserver, and Cover Glass

**Figure 2:**
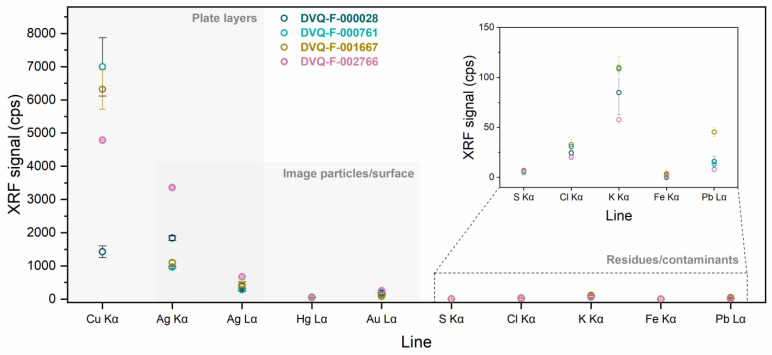
XRF results from image plates DVQ-F-000028, DVQ-F-000761, DVQ-F-001667, and DVQ-F-002667. The elements arising from plate and image particles are highlighted. The inset shows the minor elements related to residues from the manufacturing process or contaminants.

To understand how the different components in the structure of a daguerreotype interact, we characterized them first independently. The main elemental composition (i.e., Cu, Ag, Hg, and Au) of the four daguerreotypes disclosed with XRF (Figure 2) is in agreement with the typical composition reported by previous research [1,17]. The Au Lα cps suggest that all the plates studied were gilded. Some of the minor elements (Figure 2, inset) detected (e.g., Cl and K) can be associated with the manufacturing process, such as fixing and gilding. The values from the Ag (Kα and Lα) and Cu Kα lines arise from the two-layer (Cu-Ag) plate support; their variations are influenced by the inhomogeneous thickness of the Cu sheet and the Ag layer, as well as the presence of small amounts of Cu in the Ag layer [17,29]. However, because this technique offers bulk information, it is difficult to evidence the contribution of each layer from the plate.

**Figure 3:**
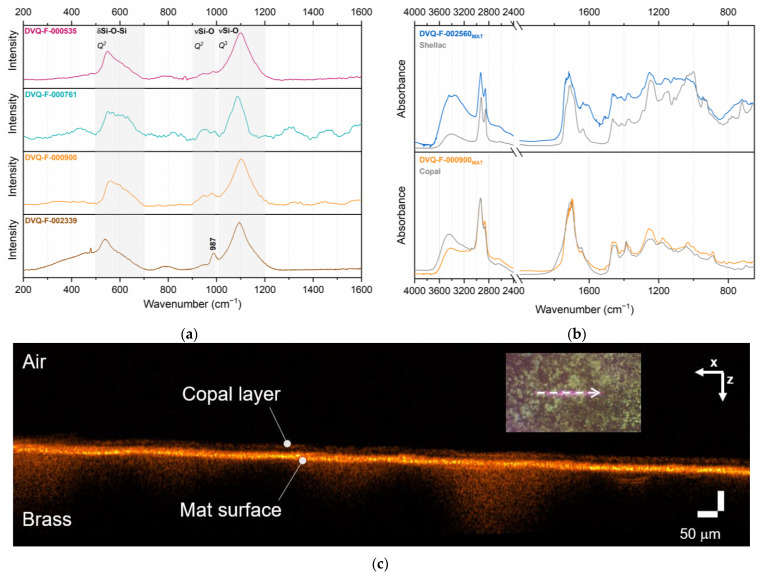
(**a**) Raman spectra from the cover glasses of the daguerreotypes DVQ-F-000535, DVQ-F-000761, DVQ-F-000900, and DVQ-F-002339. The assignment of the main bands is indicated. The sharp band at 987 cm suggests that the glass contains a small amount of Pb or Cu. (**b**) μ-rFTIR spectra of the resin layers in the mat of daguerreotype DVQ-F-002560 and DVQ-F-000900. (**c**) OCT measurement in the verso of the mat shows the resin layer in the *xz* cross-section from the profile marked with the dashed white arrow in the visible image in the inset.

Besides the image plate, other elements in the structure of the hinged case were studied. The μ-Raman spectra (Figure 3a) collected from three of the cover glasses show that they are alkali silicate glasses, which is common for the original cover glasses in daguerreotypes [24]. The spectra exhibit the typical νSi-O intense band (*Q*_3_) at 1101/1100 cm^−1^ for the glasses containing K and at 1088 cm^−1^ for those with Na. The band at 547–554 cm^−1^ can be assigned to the δSi-O-Si [30]. In particular, a band at 987 cm^−1^ was detected in the spectra obtained from the glass of DVQ-F-002339 by the McClees and Germon studio. According to Robinet et al. [30], it can be associated with low Cu or Pb (<1%) content; indeed, neither of these two elements were detected with XRF.

The XRF analysis of the metallic elements (i.e., mat and preserver) from DVQ-F-000761 confirmed the presence of an α-brass (Cu/Zn alloy) as suggested by the percentage of Zn in the mat (~11%) and the preserver (~19%) [31]. Minor constituents and contaminants, such as Pb, Sn, and Ca, were also detected. Moreover, some of the mats studied present a distinctive coloration. The results of the OCT analysis of the mat of DVQ-F-000900 show that a very thin layer, ~12.0 μm (σ = 0.9 μm), is present over the mat surface and it is not distributed homogeneously as a result of the micro-topography of the mat (Figure 3c). Under these conditions, μ-rFTIR spectra (Figure 3b) indicate the presence of two types of natural resins: shellac and copal. The spectra obtained showed features similar to the absorbance spectra, without deformations produced by the reflectance modality, probably due to the absorption-reflection phenomenon induced by the reflectivity of the metallic surface [32]. 

### 3.2. The Degradation of the Interrelated Elements

#### 3.2.1. Corrosion of Single Elements

**Figure 4:**
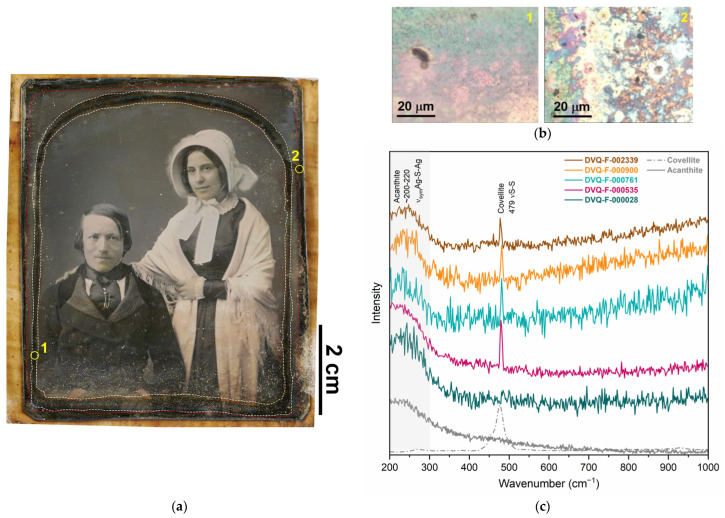
(**a**) Positive image of DVQ-F-000535, courtesy of Fondazione Alinari per la Fotografia; the edge tarnish front (red dashed line) and mat tarnish front (white edge front) are marked. The numbers indicate two regions shown under the microscope in (**b**). (**c**) Raman spectra from different daguerreotypes studied in this work, showing the broad band associated with acanthite (Ag_2_S) and covellite (CuS), compared with their respective reference spectra.

The most degraded areas of the plates were those in contact with the mat (the so-called mat tarnish front, Figure 4a) which shows the characteristic blueish dark color (Figure 4b) [10,33]. The corrosion products detected in the plates studied were sulfur compounds, mainly acanthite (Ag_2_S), as shown by the μ-Raman spectra (Figure 4c) dominated by the broad band centered at ~200–222 cm^−1^ attributed to the sum, produced by the low crystallization degree, of three bands from the ν_sym_Ag-S-Ag in Ag_2_S [34,35]. This is consistent with the typical degradation of silver in S-rich environments [36]. Additionally, in DVQ-F-000535, DVQ-F-000900, DVQ-F-001667, DVQ-F-002339, DVQ-F-002560, and DVQ-F-002694, covellite (CuS) was identified in coexistence with Ag_2_S, as suggested by the sharp peak at 479 cm^−1^ from the νS-S [34] (Figure 4c). This is in agreement with the literature reporting the tarnish front, commonly constituted by Ag_2_O and Ag_2_S [37]. Indeed, if the storage conditions are not controlled, galvanic corrosion can occur as a result of the close contact between the two metals. Hence, the degradation of daguerreotypes is more complex than the sole image plate damage, which in most cases is studied separately.

**Figure 5:**
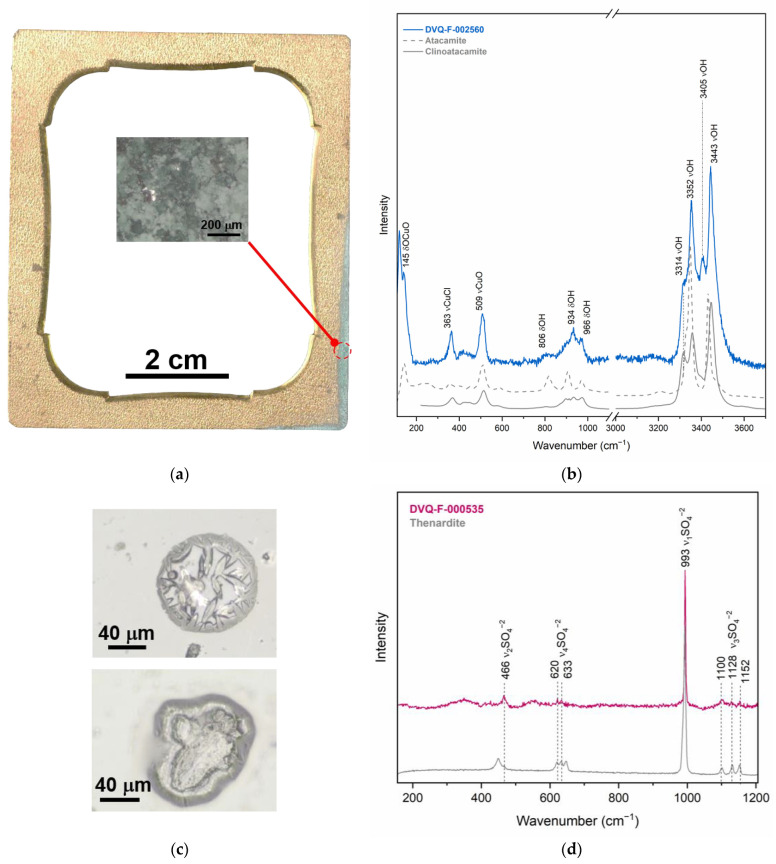
(**a**) Visible image of the mat from DVQ-F-002560, the red dashed circle indicates the area of the analysis showed in the microphotograph reported in the inset, from where the μ-Raman spectrum reported in (**b**) was obtained. (**c**) Microphotographs of two different crystals found in the cover glass of DVQ-F-000535 containing Na sulfate as suggested by the (**d**) μ-Raman spectra.

Basic copper chlorides, atacamite (Cu_2_Cl(OH)_3_)) and clinoatacamite (Cu_2_(OH)_3_Cl), were identified with Raman spectroscopy in the lower right corner of the mat of DVQ-F-002560 (Figure 5a,b). The occurrence of these two corrosion products suggests that the system is in transition to its more stable thermodynamic phase (i.e., clinoatacamite) [38]. The position and area of the damage suggest that it was produced by an external agent. Moreover, we found the same basic copper chlorides over the plate, in the area where there is direct contact between the two elements. 

Finally, some of the cover glasses showed small drop-like degradation products over the surface (Figure 5c,d). The images obtained under the microscope present some of the crystals formed over the surface of the cover glass of DVQ-F-000535, which are constituted by sodium sulfate (thenardite, α-Na_2_SO_4_-V) (Figure 5c), as indicated by the direct analysis with Raman spectroscopy [39]. This compound has been identified previously as a degradation product in stained glasses and has been correlated with Na-rich glasses exposed to the high atmospheric concentration of SO_2_ in addition to relative humidity above 65% [40]. Barger et al. [24] attributed these blister-like crystals of Na sulfate in daguerreotype cover glasses to the intentional addition of SO_2_ during glass melting to increase the stability of Na-rich glasses. Indeed, the cover glasses, despite their protective role, may have an impact on the daguerreotypes’ degradation. Nineteenth-century glasses tend to be chemically unstable, suffering from weathering [24], and tend to form Na_2_SO_4_, BaSO_4_, NH_4_Br (when Br was involved in the daguerreotype manufacturing), or HCOONa when the glass degradation products interact with the image plate surface [10,24]. Furthermore, when the glass breaks, debris can damage the plate, and the insulation against environmental gasses fails, producing corrosion that follows the shape of the fracture [37].

#### 3.2.2. Interrelated Elements: Corrosion Induced by Materials Interaction

**Figure 6:**
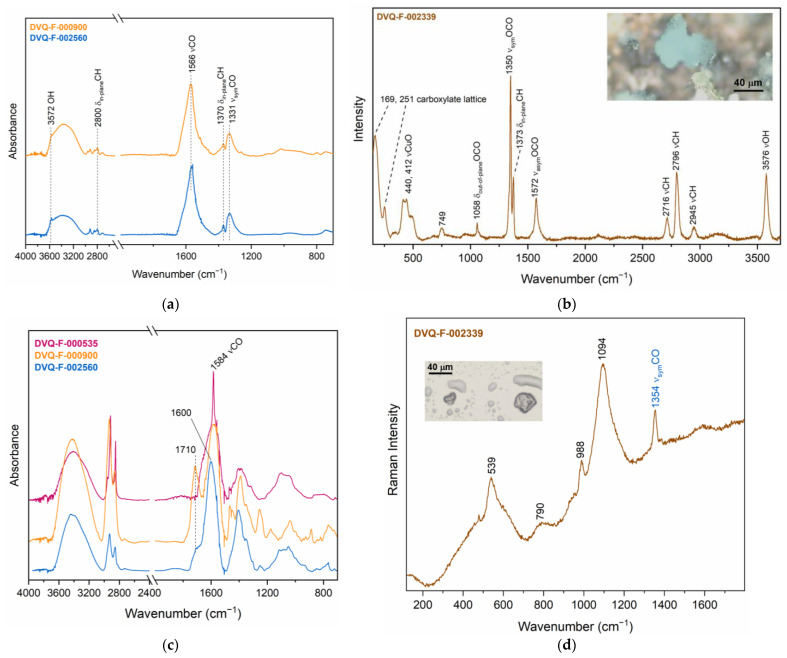
(**a**) μ-rFTIR and (**b**) μ-Raman spectra of Cu_2_(OH)_3_(HCOO) detected in the mats of DVQ-F-000900, DVQ-F-002560, and DVQ-F-002330, respectively. (**c**) μ-rFTIR of the carboxylates identified in the daguerreotypes DQV-F-000353, DVQ-F-000900, and DVQ-F-002560. (**d**) μ-Raman spectra from DVQ-F-002339, the band at 1354 cm^−1^ suggests the presence of formates (probably NaHCOO) in the glass. The inset shows the crystals formed over the glass surface.

Evidence of the interaction between the different materials was found in the various daguerreotypes studied. Macroscopically appearing dark spots are present in the majority of the mats and, in a few cases, green corrosion products were identified on a microscale in correspondence to those dark spots. The μ-rFTIR spectra (Figure 6a) from some of the dark spots evinced the presence of Cu formates, in particular, the broad band at 1596 cm^−1^ and two well-defined peaks at 1373 cm^−1^ and 1350 cm^−1^, which can be associated to dicoppertrihydroxyformate (Cu_2_(OH)_3_(HCOO)). The presence of Cu_2_(OH)_3_(HCOO) was confirmed by μ-Raman analyses (Figure 6b) in the DVQ-F-002339 mat [41,42], sometimes mixed with cuprite (Cu_2_O). 

Cu formate formation can be triggered by the interaction of metals with the emissions from organic materials, for example, wood or cellulosic fibers. Additionally, previous investigations suggest that the mechanism is induced by an alkaline environment produced by the degradation products from glass that favor the interaction of the Cu alloy with formic acid (HCOOH) derived from formaldehyde (H_2_C=O) emitted by the organic materials [43]. According to Tétreault et al. [44], formic acid has a strong effect on copper, and a very low concentration is enough to initiate corrosion reactions, producing Cu_2_O and Cu formates.

Interestingly, in addition to the formates, other carboxylate species were identified over the mats. The intense broad band at 1600–1594 cm^−1^ can be associated with the formation of metal soaps (Figure 6c), as the result of the interaction between the resins—both shellac and copal—and the metal substrate. The interaction between the metal ions from pigments (e.g., Zn white and azurite) and natural resins have been previously described in paintings and painted glasses [45,46,47]. It has been attributed to the resin aging, giving rise to the de-esterification and subsequent production of carboxylic acids that become available for complexation [46]. The capacity of brass to induce the formation of metal soaps was also detected in a painting containing brass particles, which gave rise to the formation of Cu and Zn soaps in the paint layer [48]. In DVQ-F-000535, very sharp peaks at 1584 cm^−1^, different from the broad band attributed to formates, suggest the presence of Cu carboxylate. To the best of our knowledge, this is the first time that metal carboxylates are reported in daguerreotypes, and further investigations are required to understand their formation mechanism. The results are in agreement with other corrosion products (Na-Cu formate hydroxide oxide hydrate (Cu_4_Na_4_O(HCOO)_8_(H_2_O)_4_(OH)_2_), dicoppertrihydroxyformate (Cu_2_(OH)_3_HCOO), and chalconatronite (Na_2_[Cu(CO_3_)]_2_·3H_2_O)) [41,49] detected in daguerreotypes as a result of the co-presence of glass, metals, and wood [25,49,50]. The latter emits formic and acetic acids and formaldehyde [51] that trigger degradation mechanisms in the glass and metallic elements. The glass degradation products provide the necessary pH conditions (>8) to induce further degradation mechanisms in the metallic components that produce formates [41,52]. 

Formates were found, with μ-Raman (Figure 6d), in the cover glass of DVQ-F-002339, where drop-like crystals were identified. Even though the presence of the single band at 1354 cm^−1^ is not sufficient to identify the precise composition, as reported by Robinet et al. [53], the liquid-like shape of these crystals might suggest they are K formates because they are liquid at room conditions due to their high hygroscopicity. According to Barger et al. [24], formates in glass can be correlated partially with the presence of volatile contaminants, such as formaldehyde, and CO and/or CO_2_, which may be involved in intermediate reactions under different conditions, leading to the formation of formic acid. 

**Figure 7:**
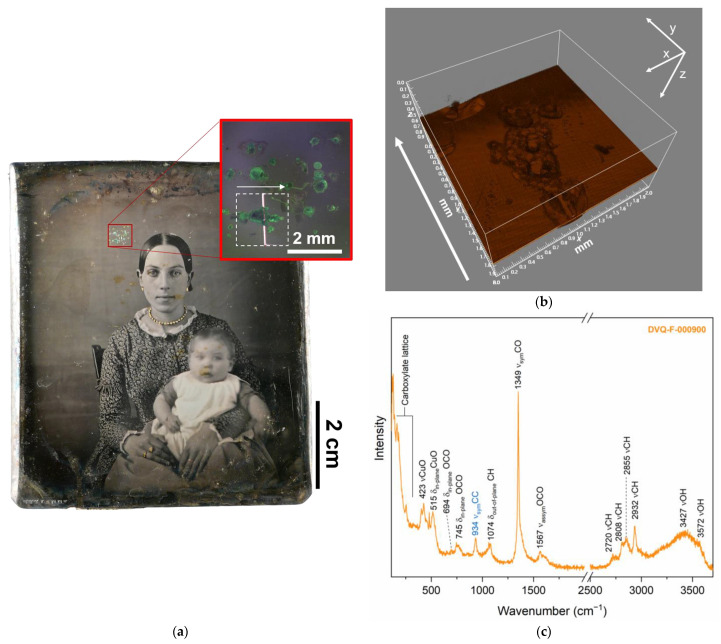
(**a**) Positive image of DVQ-F-000900, courtesy of Fondazione Alinari per la Fotografia; the red square indicates the area with formate and acetate crystals. (**b**) Sd-OCT tomocube acquired in correspondence with the white dashed square in the inset of (**a**). (**c**) Raman spectra from the green crystals, the bands associated with Cu formate (black numbers) and acetate (probably Na acetate, blue numbers).

Formates do not only affect the single elements, such as the mat or the glass, but also in DVQ-F-000900, green Cu formate drop-like crystals (Figure 7) were found to be deposited over the plate. Their position—far from other metallic elements like the mat or preserver—could suggest that Cu is derived from the plate [1,17]. The μ-Raman spectra from these green particles show similar features as those identified in the mat DVQ-F-002339 linked with Cu_2_(OH)_3_(HCOO) but differ by the presence of a band at 934 cm^−1^ that indicates the co-presence of acetate [50,54], probably Na acetate as suggested by Trentelman et al. [50]. In the 3000–3600 cm^−1^ region, the weak band at 3572 cm^−1^ can be assigned to Cu(OH)_2_ and the broad band centered at 3432 cm^−1^ suggests the presence of water [55]. 

## 4. Discussion

As the results show (Table 3), the conservation of a daguerreotype image goes beyond the single image plate. Their structural complexity and the multiplicity of materials present must be considered. Daguerreotypes behave as an interconnected and partially closed system where factors, such as humidity and volatile pollutants, are particularly important. 

The interaction between the different materials is complex and some mechanisms still require further investigation, for example, the formation of metal carboxylates in the presence of natural resins. The presence of organic volatile compounds (VOCs) plays a particular role in the degradation of the whole structure, yet the currently available preservation guidelines do not include the monitoring of VOCs during daguerreotype storage. The main factors considered are relative humidity (best 30–35%), temperature (<24 °C), and light exposure (86–130 lux). Therefore, the development of fast and easy VOCs monitoring strategies available for conservators would be of utmost importance and utility. 

## 5. Conclusions

The proposed multi-modal protocol, exploiting elemental (i.e., XRF) and molecular spectroscopy (i.e., μ−Raman and μ−rFTIR) combined with OCT, allowed disclosing a wide range of degradation products present in the daguerreotypes from the Fondazione Alinari per la Fotografia (Florence, Italy) collection in a non-invasive way. The combination of bulk elemental information, molecular data obtained at a microscopic scale, and the morphological data (i.e., OCT) allowed for robust and complementary identification of the composition and degradation of the complete structure of the objects as a correlated system. Given the micrometric size of the corrosion products, the high spatial and spectral resolution of the employed technologies proved fundamental. 

Particularly important is also the high specificity of the spectroscopic molecular techniques. Given the typology of the object, the Raman signal was not hindered by the fluorescence emission. Its high spatial resolution (down to 1 micron) and accessibility to the wavenumber region allowed this sensing technology to provide very comprehensive results. The intrinsic characteristics of the daguerreotype materials enabled to obtain results with an adequate signal-to-noise ratio reflectance FTIR spectra, mainly due to the high reflectivity of the metallic surfaces (i.e., the mirror-like image plate and mat surface). This eliminates the need for direct contact of the examined surface with the FTIR probe. The main evidence of the correlated degradation, provided by Raman, FTIR, and OCT, is the copper formates, present both in the mat and the plate, resulting from the interaction between the glass, the case, and the metallic elements. In this respect, our approach shows the complementary different optical instrumentation that allows registering the morphological characteristics of the materials and degradation by-products, together with the chemical composition. Some other products, not previously reported for daguerreotypes, such as Cu carboxylates caused by the resin and metals in the mat interaction, were also detected. 

The results demonstrate the validity of the proposed analytical protocol to reveal the interaction between all the components present in their structure. The degradation is generally caused by unfavorable atmospheric conditions (high RH, presence of VOC, and S/Cl-containing pollutants) and secondarily by the interaction of the different elements composing the structure of the daguerreotypes. These phenomena must alert conservators in charge of the preservation of daguerreotype collections and draw attention to the development of effective monitoring strategies for preventive conservation. 

## Figures and Tables

**Table 2:** Daguerreotypes studied in this work. The colors next to the codes are used in the figures of this work to associate the relevant data with the examined object.

Code *	Subject	Plate Dimensions (cm^2^)	Historical Information [27,28]
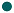 DVQ-F-000028	Portrait of Zelinda Tallini in Galli	10.7 × 8.1	ca. 1846–1848, Claude Porraz (Bologna, Italy).
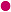 DVQ-F-000535	Couple portrait	7.60 × 6.40	Before 1845. Unidentified author.
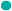 DVQ-F-000761	Portrait of a girl	8.4 × 7.2	After 1850. Unidentified author. Hallmark: Asterisk J P Doublé 40. Probably a French plate maker (production from ca. 1850–1858).
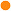 DVQ-F-000900	Portrait of a woman with a child	7.9 × 6.9	ca. 1840–1850. Unidentified author.
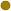 DVQ-F-001667	Portrait of a young woman	11.0 × 8.5	ca. 1855, Désiré François Millet (active in London, UK).
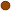 DVQ-F-002339	Male portrait	14.0 × 10.7	1847–1860, James E. McClees and Washington Lafayette Germon studio (Philadelphia, PA, USA).
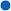 DVQ-F-002560	Male portrait in half-length	7.7 × 6.9	Before 1845. Unidentified author.
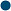 DVQ-F-002694	Portrait of a seated man with a cane	8.2 × 7.1	After 1850. Unidentified author.
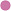 DVQ-F-002766	Female portrait	7.9 × 6.4	ca. 1841–1851. Antoine François-Jean Claudet (active in London, UK).

* Code corresponds to the ID number from FAF. Codes can be used to search the daguerreotypes in the FAF Unique Object collection available online [27].

**Table 3:** Summary of the degradation products identified in this work and the possible factors involved in their formation.

Daguerreotype Element	Degradation Product	Analytical Technique	Factors Involved
Cover glass (analyzed with XRF, μ-Raman)	Na sulfate (Na_2_SO_4_)	μ-Raman	S-containing pollutants
	Formates	μ-Raman	VOCs from the case and relative humidity
Mat (analyzed with XRF, OCT)	Atacamite (Cu_2_Cl(OH)_3_), clinoatacamite (Cu_2_(OH)_3_Cl)	μ-Raman	Chlorine contamination
	Cu formate, cuprite (CuO)	μ-Raman, μ-rFTIR	VOCs from the case and relative humidity
	Cu carboxylates	μ-rFTIR	Resin–metal interaction, relative humidity, VOCs from the case?
Image plate (analyzed with XRF, μ-Raman, OCT)	Acanthite (Ag_2_S)	μ-Raman	S-containing pollutants
	Covellite (CuS)	μ-Raman	S-containing pollutants
	Atacamite (Cu_2_Cl(OH)_3_)	μ-Raman	Degradation products from the mat
	Cu formate, Cu acetate	μ-Raman, OCT	Degradation products from glass, VOCs from the case, relative humidity

## Data Availability

Data available on reasonable request to the corresponding author.
